# Cognitive and Experienced Flexibility in Patients With Anorexia Nervosa and Obsessive Compulsive Disorder

**DOI:** 10.3389/fpsyt.2022.868921

**Published:** 2022-05-09

**Authors:** Lot Catharina Sternheim, Boris van Passel, Alexandra Dingemans, Danielle Cath, Unna Nora Danner

**Affiliations:** ^1^Department of Clinical Psychology, Utrecht University, Utrecht, Netherlands; ^2^Centre for Anxiety Disorders Overwaal, Institution for Integrated Mental Health Care, Pro Persona, Nijmegen, Netherlands; ^3^Behavioural Science Institute, Radboud University, Nijmegen, Netherlands; ^4^Rivierduinen Eating Disorders Ursula, Leiden, Netherlands; ^5^Department of Specialist Training, GGz Drenthe, Groningen, Netherlands; ^6^Department of Psychiatry, Rijksuniversiteit Groningen and University Medical Center Groningen, Groningen, Netherlands; ^7^Altrecht Eating Disorders Rintveld, Zeist, Netherlands

**Keywords:** OCD, AN, set-shifting, detail focus, cognitive flexibility

## Abstract

**Objective:**

Anorexia nervosa (AN) and obsessive-compulsive disorder (OCD) share a neuropsychological profile characterized by cognitive inflexibility as evident in set-shifting problems, and by strong detail focus. Clinically, both patient groups display a strong rigidity which may be explained by these neurocognitive difficulties. Cognitive inflexibility may hinder treatment uptake and help explain suboptimal treatment outcomes in both AN and OCD. This is the first study to compare clinical AN and OCD groups andto examine similarities and differences in cognitive flexibility. Specifically, this study aims to investigate neuropsychological outcomes and self-reported difficulties in both clinical groups and a control group, and explore associations between the different flexibility outcomes and illness.

**Method:**

Two hundred participants (61 AN, 72 OCD and 67 HC) performed neuropsychological tasks on set-shifting abilities (Trail Making Task, Stroop color-word interference, Intradimensional-Extradimensional shift task), detail focus (Group Embedded Figures Test) and self-reported set-shifting abilities and attention to detail (DFlex).

**Results:**

Similarities between patient groups were found in terms of reduced set-shifting ability on the Trail Making Task and detail focus. Moreover, both patient groups self-reported more set-shifting problems but a less strong detail focus than HC, which in turn were not related to neuropsychological task outcomes in either of the groups. In both patient groups longer illness duration was associated to longer reaction times in the switching tasks and for both groups symptom severity was associated to higher experienced inflexibility and attention to detail.

**Conclusion:**

Cognitive inflexibility processes are largely similar in patients with AN and OCD. Both patient groups report inflexibility, yet this is unrelated to neuropsychological outcomes. Illness duration seems to contribute to poorer set-shifting and higher illness severity is linked to more experienced inflexibility. Findings highlight the need for entangling different domains of cognitive flexibility and detail focus and examining self-report measures for a cohesive understanding of clinically relevant flexibility weaknesses in AN and OCD.

## Introduction

High rates of comorbidity between obsessive-compulsive disorder (OCD) and anorexia nervosa (AN) have been reported, with frequencies of OCD in patients with AN being between 9.5 and 62% (1, 2) and frequencies of AN in 11–42% in patients with a primary diagnosis of OCD (1, 3). This high comorbidity may be the consequence of strong genetic correlations between the two (4) and is also evident phenomenologically, as characterized by repetitive and compulsive ritualistic behavior, difficulties in set-shifting and obsessive worrying (5, 6).

However, to the best of our knowledge, few studies have directly compared neuro- psychological test performance of patients with OCD with that of patients with AN, to investigate potential shared neuropsychological impairments (7). No study has compared patients with OCD and with AN on cognitive flexibility, either on neuropsychological level or experienced flexibility, within one study design. In both patient populations separately, studies have focused on two key components of cognitive flexibility, namely set-shifting ability and central coherence strength. Several studies reported comparable set-shifting inefficiencies (8, 9) and central coherence weaknesses (10, 11). Several studies reported comparable set-shifting inefficiencies in OCD and AN (8, 9) and central coherence weaknesses (10, 11).

In AN, a substantial amount of studies, reviews and even a meta- review found set-shifting difficulties in adults with AN (12–14). Moreover, both meta-analyses and systematic reviews show inefficiencies in global processing in combination with a greater propensity for detail-focused processing in adults with AN (13–15). In OCD set-shifting has been extensively researched with three meta-analyses (16–18) reporting moderate effect sizes for shifting problems and moderate effect sizes for visuospatial abilities.

This common rigid neuropsychological profile of set-shifting inefficiencies and detail focus (vs. global processing), may be driven by similar dysfunctional brain behavior pathways (19). This in turn may explain the high levels of comorbidity across the two disorders. Evidence for this shared profile is found in studies in patients with AN highlighting relations between cognitive flexibility and OCD symptoms. For example, impaired set-shifting and aspects of central coherence (i.e., strong detail focus) as measured with neuropsychological tasks (20) but also self-reported strong detail focus (21) were found to be associated with more symptoms of OCD in patients with AN. Levinson and colleagues (22, 23) tested which cognitive-behavioral aspects of OCD are most relevant in AN and highlighted the relevance of concerns over mistakes and obsessions as potential shared feature between OCD and AN.

OCD patients also report difficulties in attention switching and elevated attention to detail (24) and similar difficulties have been found in a sub-clinical OCD group (25). The study by Sternheim and colleagues (25) furthermore identified a lack of associations between a neuropsychological measure of cognitive flexibility and experienced flexibility as measured using self-report instruments. Interestingly, there are as yet no studies in AN or OCD that investigate associations between neuropsychological outcomes and experienced cognitive flexibility.

Studies directly comparing clinical AN and OCD groups are needed to understand differences and similarities in cognitive flexibility which in turn may provide insight into the shared clinical features of AN and OCD, help understand the high comorbidity of AN and OCD and inform treatment for both AN and OCD. The first aim of this study thus is to investigate potential differences and similarities in cognitive flexibility Secondly, this study examines relations between neuropsychological flexibility outcomes and self-report outcomes. This is in line with recommendations following a recent systematic review on cognitive flexibility and (aspects of) central coherence in AN (such as attention to detail) (9). This review highlights that individuals with AN report themselves to be more inflexible compared to the general population and recommends integrating self-report measures into assessments of cognitive flexibility (9).

The first aim of this study was to explore similarities and differences in set-shifting and detail focus in a relatively large clinical sample of individuals with AN and OCD, and compare the groups to healthy controls (HC). We expected both patient groups to show similar inefficiencies on neuropsychological tasks assessing set-shifting, show a stronger detail focus, and report less experienced flexibility, when compared to HC. Our second aim was to investigate associations between the neuropsychological tasks and a self-report measure across the two patient groups. Thirdly, we examined associations between neuropsychological and experienced flexibility outcomes and illness severity in the patient groups.

## Method

The data were collected at the baseline assessment of a randomized controlled trial testing the effectiveness of Cognitive Remediation Therapy in patients with OCD and AN (26). The trial was approved by the medical ethics committee of the University Medical Center Utrecht [METC no. NL43751.041.13 v.03)] and registered at the Netherlands Trial Register (NTR3865) prior to the start of data collection.

## Participants

In total, 61 patients with AN (AN-R = 42, AN-BP = 16, AN but unknown subtype = 3), 72 patients with OCD and 67 HC participants were included in the study. The patients were recruited from four highly specialized treatment centers in the Netherlands. Inclusion criteria for the study included: age 18–60 years, fulfilling DSM-IV-TR criteria for AN (or an eating disorder not otherwise specified clinically referred to as AN), or OCD. Exclusion criteria were: neurological illness, comorbid psychiatric disorder if severity hindered study participation (as assessed by psychiatrists or clinical psychologists), intellectual impairment, and inability to adequately speak or read Dutch. Stable doses of antidepressants and antipsychotics were allowed. Benzodiazepines were only allowed when used as sleep medication. For a flow diagram of inclusion, information about medication and for other detailed information, see [van Passel and colleagues (26)].

HCs were recruited at the University of Utrecht, using flyers and social media advertising and were matched as much as possible with the patients groups on age and gender. However, patient groups did not match on age and sex (i.e., OCD groups inherently has an older population and more men than AN groups), and therefore we were unable to fully match all 3 groups. Inclusion criterion was BMI between 18.5 and 25 kg/m^2^ and participants who reported a lifetime or current psychiatric condition were excluded. Written informed consent was obtained from all participants.

## Measurements

### Demographic and Diagnostic Information

For all participants information on gender, age, years of education and weight was collected, and additionally information was collected on age of onset and illness duration for the patient-groups. In the patient-groups, primary diagnosis was confirmed with the Structured Clinical Interview on DSM-IV axis I disorders (SCID-I) (27). Psychopathology in the HC-group was checked with the Mini International Neuropsychiatric Interview (28). The Dutch version of the National Adult Reading Test (DART) (29) was used as a measure of premorbid intelligence level. Eating disorder severity over a 28-day period was assessed using the 36-item Eating Disorder Examination Questionnaire (EDEQ) (30). OCD symptom severity was assessed using the Yale-Brown Obsessive-compulsive Scale (Y-BOCS) (31).

### Neuropsychological Tests

To measure set-shifting the following instruments were used: (1) the digital version of the Trail Making Test (TMT, part B reaction time in msec) (32, 33), (2) the digital version of the Intradimensional-Extradimensional shift task (ID/EDS). Outcomes included the number of people that failed to reach state 9 and the average number of trials to reach stage 9 (i.e., the ED switch-cost) (34), and (3) the paper-and-pencil version of the Color Word Interference Test (CWIT) (reaction times in sec and number of errors) of the Delis-Kaplan Executive Function System (D-KEFS) (35). To measure strength in detail focus we used the Group Embedded Figures Test (GEFT) (36) which is a paper-and-pencil task. Additionally, the Detail and Flexibility Questionnaire (DFlex) (including the Cognitive Flexibility and Attention to Detail subscales) (37) assessed the self-reported experience of flexibility. The Cronbach's alpha for the Cognitive Flexibility and Attention to Detail subscales were excellent, 0.90 and 0.91, respectively. The English version of the DFlex was translated, into Dutch back translated to English and checked by the researchers of this study. Whilst no Dutch validation study has yet been completed, a French validation study concludes good psychometric properties of the DFlex (38). A more elaborate description of these measures can be found in van Passel and colleagues (39).

All participants were given the same order of tasks and questionnaire measures.

## Data Analyses

To test differences in cognitive flexibility between the groups, univariate ANCOVAs were conducted with the neuropsychological outcomes and the self-report outcomes as the dependent variable and group (AN, OCD, HC) as the predictor variable adjusting for age. Violation of assumptions and adjustments resulting therefrom are described per measurement, see Table 1. Partial correlations were calculated adjusting for age to test relations between the neuropsychological and self-report measures for each patient group separately as well as for the two patient groups together. Partial correlations adjusting for age were also calculated to test relations between neuropsychological and self-report measures and illness severity for each patient group separately (i.e., BMI and EDEQ scores for the AN group and YBOCS score for the OCD group).

**Table 1:** Mean, SD and between group differences for demographic, clinical and flexibility variables of all groups.

	**AN (*n* = 61) M (SD)**	**OCD (*n* = 72) M (SD)**	**HC (*n* = 67) M (SD)**	**Results**		
				***F/X^**2**^***	***p***	*** $\eta_{p}^{2}$ ***
Demographic and clinical characteristics						
Age in years	24.90 (7.3)	33.92 (10.9)	29.21 (11.3)	13.27	<0.001	0.12
Education Years	14.47 (2.0)	13.23 (4.0)	18.64 (3.5)	38.53	<0.001	0.36
DART score	83.93 (10.6)	82.46 (14.2)	85.23 (10.0)	0.93	0.40	—
8ptAge of onset in years	16.88 (4.8)	18.11 (7.9)	—	1.05	0.31	—
8ptIllness duration in years	4.88 (6.1)	8.91 (11.2)	—	6.06	0.015	0.05
BMI in kg/m^2^	16.06 (1.8)	24.21 (4.5)	22.47 (3.3)	99.06	<0.001	0.51
EDEQ	3.86 (1.2)	1.24 (1.4)	0.73 (0.9)	116.41	<0.001	0.56
Y-BOCS	5.95 (9.2)	23.91 (6.5)	0.21 (0.6)	237.92	<0.001	0.71
Cognitive and subjective flexibility (adjusting for age)						
TMT^†^, ^‡^ in ms	3,707.67 (1,231.83)	4,516.45 (2,614.63)	3,342.36 (914.62)	5.26	0.006	0.05
ID/EDS^§^						
10ptNo. people stage 9 not reached	21	27	23	0.20	0.91	—
10ptNo. trials to reach stage 9 (ED switch-cost)	16.51 (9.30)	15.26 (11.60)	17.39 (10.71)	0.52	0.60	0.01
CWIT-rigidity^†^, ^¶^						
Time in sec	52.73 (8.92)	58.98 (16.36)	53.03 (12.17)	1.77	0.17	0.02
Error	1.25 (1.47)	1.01 (1.47)	0.98 (1.09)	1.20	0.30	0.01
GEFT^††^, ^‡‡^						
No. errors	1.68 (2.04)	3.35 (3.42)	0.46 (1.05)	20.52	<0.001	0.18
Time in sec	173.01 (62.33)	190.80 (70.26)	No data	1.74	0.19	0.01
DFlex						
Cognitive rigidity	44.48 (11.10)	43.54 (12.76)	27.04 (7.17)	55.35	<0.001	0.37
Attention to detail	38.72 (11.73)	40.99 (12.97)	24.85 (8.48)	39.57	<0.001	0.30

*AN, Anorexia Nervosa; OCD, Obsessive-compulsive disorder; HC, Healthy Controls; DART, Dutch adult reading test; BMI, Body mass index; EDEQ, Eating Disorder Examination Questionnaire; YBOCS, Yale-Brown Obsessive Compulsive Scale; TMT, Trail Making Test; ms, milliseconds; IDE/EDS, Intradimensional/Extradimensional Shift-task; CWIT-rigidity, Color Word Interference Test – rigidity part; GEFT, Group Embedded Figure Test; DFlex, Detail and Flexibility Questionnaire*.

† *TMT mean scores and CWIT-rigidity time scores were log transformed; mean and SD scores presented are the raw scores. Outliers were identified and omitted when scores were more than 3x SD from the specific group mean*.

‡ *Effect of adjusting variable age: F (1.189) = 45.06, p < 0.001, η_p_^2^ = 0.19*.

§ *Two outliers in the OCD-group were still present after log transformation of the time-scores. These were removed before the analyses and from the raw scores that are presented. Effect of age variable: F (1.191) = 11.95, p < 0.001, $\eta_{\text{p}}^{2}$ = 0.06*.

¶ *Two outliers were removed before running the analyses. Effect of age variable: F (1.191) = 1.79, p = 0.18, $\eta_{\text{p}}^{2}$ = 0.01*.

†† *For the number of errors, in both OCD and HC group, two outliers were identified, these were removed before running the analyses. Effect of adjusting variable age: F (1.190) = 8.28, p = 0.004, $\eta_{\text{p}}^{2}$ = 0.04*.

‡‡ *For the time scores, in the OCD-group one outlier was identified who was removed before the analysis. The adjusting variable was non-significant*.

*^§§^Adjusting variable age in both analyses was non-significant*.

## Results

### Participants Characteristics

The general demographic and clinical information (see Table 1) showed that the three groups differed in age. Since, the two patient groups also differed in illness duration, but not in age of onset, age was added as a co-variate to the analyses to be able to include the control group in the analyses. Results further revealed that HC-participants had more education years compared to both patient groups (these last groups did not differ), but there were no between group differences regarding their premorbid intelligence level (as reflected by the Dart scores). The results of the clinical outcomes (BMI, EDEQ and YBOCS) were as expected. Patients with comorbid AN and OCD diagnoses were included into the study, analyses confirmed no differences in outcomes when excluding these participants (*n* = 12).

### Neuropsychological Outcomes

All between-group effects are reported in Table 1.

#### Set-Shifting

The three groups differed regarding their average reaction times on the TMT. Pairwise comparisons showed that the AN- and OCD-groups had longer reaction times, compared to the HC-group, resp. *p* = 0.014 and *p* = 0.003, suggestive of set-shifting impairments, but they did not differ from each other, *p* = 0.69. No between-group differences on the ID/EDS were found for the number of people that failed to reach state 9 and 2 the average number of trials to reach stage 9 (i.e., the ED switch-cost). Moreover, no between-group differences were found on the average reaction times and number of errors of the CWIT.

#### Detail Focus

With regard to the GEFT a significant between-group difference was found regarding the total number of errors that were made. Pairwise comparisons showed that the three groups differed significantly from each other, with OCD-patients making the most errors in detecting the smaller shapes in the larger shapes compared to both AN, *p* = 0.006, and HC, *p* < 0.001, suggesting that OCD-patients had the weakest focus to detail, with the AN group scoring in between OCD and HC, *p* = 0.001, and with the HC-participants making the least errors. Of note, in the HC-group as a result of problems with the administration of the GEFT, no data on the GEFT time variable were available. Results regarding the total time without errors did not differ between the two patient-groups.

### Self-Report Outcomes

There were between-group differences for both subscales of the DFlex. Pairwise comparisons showed that patients with AN and OCD reported similarly increased experiences of cognitive inflexibility, *p* = 0.85, and of attention to detail, *p* = 0.34, in comparison to the control participants, both *p*'s < 0.001.

### Correlations Within and Across Patient Groups

Partial correlation analyses adjusting for age are presented in Table 2 for each patient group separately and for the two patients groups combined. Outcomes showed that no relations were found between the outcomes on the two DFlex subscales and any of the neuropsychological tasks.

**Table 2:** Partial correlations adjusting for age for the anorexia nervosa and obsessive compulsive disorder groups separately and together between the self-report measures of flexibility (DFlex) and the different set-shifting (TMT and CWIT) and central coherence (GEFT) measures.

		**TMT time (ms)**	**CWIT time**	**CWIT error**	**GEFT error**	**GEFT time**		
Anorexia nervosa
DFlex rigidity	*r*	0.25	−0.09	0.16	0.02	−0.16		
DFlex attention	*r*	0.09	−0.09	0.24	0.15	−0.04		
							DFlex rigidity	DFlex attention
Illness duration	*r*	0.11	0.35^**^	−0.01	−0.23	0.25	0.21	0.18
BMI	*r*	0.22	0.10	0.22	0.10	−0.09	0.03	−0.11
EDEQ	*r*	0.22	−0.12	0.18	0.03	−0.03	0.41^***^	0.32^*^
Obsessive compulsive disorder
DFlex rigidity	*r*	0.15	0.17	0.05	−0.05	0.07		
DFlex attention	*r*	0.14	0.20	0.04	−0.10	0.11		
							DFlex rigidity	DFlex attention
Illness duration	*r*	0.34^*^	0.37^*^	0.12	0.11	0.10	0.02	0.12
BMI	*r*	−0.06	0.08	0.01	0.14	−0.01	0.02	−0.17
Ybocs	*r*	0.17	0.24	0.10	0.26	0.07	0.22	0.43^**^
Across patient groups
DFlex rigidity	*r*	0.17	0.11	0.08	−0.03	0.01		
DFlex attention	*r*	0.12	0.13	0.10	−0.02	0.07		

*For the two patient groups separately, additional partial correlations adjusting for age were also shown between all measures and clinical outcomes (BMI in kg/m^2^, illness duration and severity of the pathology with EDEQ scores for the AN group and YBOCS scores for the OCD group)*.

* *p < 0.05*,

** *p < 0.01*,

*** *p < 0.001*.

*TMT, Trail Making Test; ms, milliseconds; CWIT, Color Word Interference Test–rigidity part; GEFT, Group Embedded Figure Test; DFlex, Detail and Flexibility Questionnaire*.

For the AN group, longer illness duration was associated to longer reaction times on the CWIT. Higher eating disorder pathology as measured with the EDE-Q was associated to higher experienced inflexibility and attention to detail.

For the OCD group, longer illness duration was associated to longer reaction times on both the CWIT and the TMT. Higher OCD pathology (as measures with the Y-BOCS) was associated to higher experienced attention to detail.

## Discussion

In sum, whilst there were some differences between the OCD and AN groups, the groups showed overall similar levels of cognitive inflexibility, corroborating the clinical features observed in both groups and in line with previous studies in each of the two groups. Specifically, patients with AN and OCD showed similarly increased reaction times on the TMT, reflecting set-shifting impairments, as compared to HC confirming hypotheses with a medium effect. Contrary to expectations we found no set-shifting impairments for the AN and OCD groups on the other two set-shifting measures (i.e., ID/EDS and CWIT-rigidity). Similarly, against our expectations we found that the HC group showed the strongest focus to detail instead of the AN or OCD groups. Patients with AN and OCD differed on the GEFT, with OCD patients making the most errors on the GEFT and thus displayed the weakest detail focus (with a large effect). Additionally, both patient groups reported comparably elevated levels of experienced cognitive inflexibility, with large effect sizes. There were no associations between the neuropsychological tasks and self-report measure in either patient group.

For the AN group, illness duration was associated to reaction times on the neuropsychological set-shifting measures and eating disorder pathology was associated to both experienced inflexibility and attention to detail. For the OCD group illness duration was associated to reaction times on the CWIT and OCD symptoms were associated to experienced inflexibility and attention to detail. In sum, whilst there were some differences between the OCD and AN groups, the groups overall showed similar patterns of cognitive inflexibly.

For the OCD group these findings only partly confirm those in the meta-analysis by Snyder and colleagues (18) who found elevated reaction times on CWIT-rigidity, ID/EDS as well as TMT while in our study, increased reaction times were found only on the TMT. Snyder et al. (18) and Abramowitch (16) also found inefficiencies in visuospatial abilities, which is, arguably, in line with the increased number of errors on the GEFT. For the AN group, the current findings in large fit with the main conclusions from a recent systematic review by Miles and colleagues (9), which identifies mixed results regarding cognitive inflexibility in adults with AN. The review highlights that, with 30 different measures used for assessing cognitive flexibility, numerous versions of a task ánd multiple outcome measures within a task, it is challenging to compare findings.

It has moreover been argued that different set-shifting measures target multiple domains of cognitive flexibility (e.g., attention and learning processes) (40). This may explain varying outcomes on the different set-shifting measures and highlights the need for the development of neuropsychological tasks that entangle the different domains and identify those that patients with AN and OCD struggle with. This may also help explain our unexpected GEFT outcomes. Interestingly, two other studies using an Embedded Figure Task (41, 42) found behavioral results in line with ours (i.e., more errors and longer reaction times compared to HC) and in contrast to those generally found in AN (i.e., strong detail focus and poor global integration in adults with AN (14, 15). Of note, these studies used fMRI versions of the task which in itself make a direct comparison with our study results difficult. All in all, in general it appears that different methodologies or even different versions of a same task assessing the supposedly same phenomenon may provide different outcomes. This suggests very specific pathways to the behaviors we observe clinically, and a better understanding of these pathways and the extent to which different tasks assess them is required.

However, because response time data for the HC-group on the GEFT are missing, conclusions remain speculative.

Interesting is our finding that experienced inflexibility was not only much higher in the patient groups compared to the HC group, but also that higher levels of experienced inflexibility were associated to more illness severity. It may be that the experience of inflexibility, and the associated distress, is such a burden on the actual cognitive flexibility that it hampers executive functioning. Indeed, the hypothesis that cognitive and emotional processes may interfere with cognitive functioning has been posited before. For example, Gross (43) posits that emotion regulation requires cognitive resources such as selfmonitoring and self-corrective actions, which in turn reduce the resources available for other cognitive processes (such as memory and task switching). Indeed, both patients with AN and OCD are thought to have high levels of negative emotions alongside emotion regulation difficulties (44–46). It is also imaginable that negative thoughts such as worries and obsessive thoughts, both relevant to AN and OCD, may simply “fill up” the cognitive space available for patients which results in less available cognitive tools to navigate situations that require flexibility.

An alternative explanation for lack of differences between AN, OCD and HC groups on some. of the set-shifting task is that the neuropsychological tasks may not capture the rigidity as reported by patients and observed by clinicians. As also recommended by Miles and colleagues (9), we included a self-report measure for cognitive flexibility seeing that self-report measures may provide important insights into the observed and experienced inflexibility above and beyond neuropsychological tasks. Indeed, in line with previous studies, both patients groups reported comparably elevated levels of experienced inflexibility (24, 25, 47).

However, these self-reported levels of experienced cognitive flexibility did not correlate to the tasks outcomes, suggesting a discrepancy between the actual (neuro-related) switching abilities patients have, and their experienced abilities. -Difficulties being flexible and the distress associated with change we see in the clinical setting may not lie with an actual inability, but rather with the cognitive costs of regulating emotional states or negative thoughts or with negative beliefs about their abilities. Similarly, Sternheim and colleagues (48) found that whilst AN patients were able to generate an effective social problem strategy, they also described rather engaging in avoidant and socially-submissive behaviors due to low confidence in social problem solving skills. Clinically, this suggests that training cognitive skills is not enough, but that attention should be given to underlying beliefs about these skills and emotion regulation strategies.

This discrepancy highlights the difficulty to capture clinically relevant inflexibility into research assessments. One issue may be that cognitive inflexibility studies, including the current one, tend to assess very basic and “cold” neuropsychological processes in lab settings. These are often void of contextual factors, i.e., obsessive thoughts or strong negative emotions [which we know affect neuropsychological functioning; (43)], as these tend not to be captured in the neuropsychological tasks (49, 50). Possibly the reported and observed rigidity we are trying to tease out is context-dependent, surfacing in situations that entail disorder-related stimuli (i.e., food, weight for AN; intrusions for OCD) or strong negative emotions. Future studies should focus on these contextual factors by developing more ecologically valid and experimental paradigms. Indeed, Miles and colleagues (9) describe a need for a “comprehensive and cohesive understanding of cognitive flexibility” (p2), e.g., with qualitative research. In line with this future studies may benefit from incorporating a measure that assesses central coherence more completely.

Looking at associations between flexibility outcomes and clinical variables, it seems that illness duration is particularly associated to flexibility as measured with reaction times. For both patient groups, longer illness duration was associated to longer reaction times, suggesting that the longer they are ill the slower they respond. In line with a recent systematic review in AN (51), the neuropsychological outcomes were not associated to BMI. Interestingly, the self-report measures were also not associated to BMI, whilst they were associated to eating pathology as measured with the EDE-Q. Findings suggest that individuals with more or more severe symptoms of the disorder describe themselves as more rigid and more detail focused. In OCD, one study (52) suggests that slower performance on a task-switching procedure is related to higher symptom severity, but leads to more accuracy (decreased errors) which might represent a strategic tradeoff for the sake of accuracy. In two meta-analyses, symptom severity did not moderate effect sizes on executive function (16, 18). Nakao et al. (53) found longer reaction times on the Stroop-Test in a group with longer illness duration as compared a group with shorter illness duration.

Seeing that poor cognitive flexibility is thought to contribute to the maintenance of symptoms, and contribute to treatment resistance (54), entangling different components of cognitive inflexibility is crucial for the development of intervention strategies. Following our findings, treatments should address patient's experienced inflexibility and challenge beliefs regarding their ability to adapt and change. Namely, if an individual experiences a situation requiring flexibility, yet doubts their abilities, training cognitive skills may not be sufficient and additional clinical interventions increasing self-efficacy may be required.

Secondly, seeing that flexibility may be strongly context related, treatment may need to focus on providing patients with tools to manage these situational factors (i.e., increase emotion regulation skills). It is also possible that difficulties arise in the translation process from skills to execution.

Limitations of this study include the suboptimal matching of patients and controls and of the patient groups among each other, on age and education level. Despite adjusting for age in the analyses, older age of the OCD group might still have (indirectly) contributed to between-group differences on the TMT. However, the ages of these groups represent clinical reality and in fact the age differences are still relatively small. With the GEFT reaction times being unavailable we cannot compare these to previous studies using the GEFT. Whilst this limits the salience of our conclusions, we did have data on errors, in line with other studies, and we did report these. We have not excluded patients with comorbid AN and OCD diagnoses and although results did not change, this may obscure any disorder-specific effects. Future studies would also benefit from including an AN-OCD group to further study the similarities and differences between the two patient populations. A review on reviews on neuropsychological functioning in eating disorders concluded that there is currently no evidence of significant differences between AN subtypes (14). However, this review also highlights that there are a couple of studies that do find differences on set-shifting measures and therefore outcomes are somewhat mixed. Future studies should examine these differences.

Taken together, this is a first study that has directly compared OCD- and AN-patients on measures of switching, and detail focus and experienced cognitive flexibility. The results highlight some shared inefficiencies across AN and OCD groups, namely set-shifting difficulties on the TMT and poor experienced cognitive flexibility. This suggests potential benefits of trainings like Cognitive Remediation Therapy (55) where patients are offered strategies to manage these challenging situations, for patients with AN and OCD. A version of CRT that includes training emotional skills may also be useful (CREST; Tchanturia et al., (56). Moreover, findings highlight that despite clear clinical relevance of inflexibility, assessment of these processes is complex and requires attention. Importantly, this study highlights the relevance of high levels of experienced inflexibility in both AN and OCD, suggesting that including a training for increasing self-efficacy related to situations requiring flexibility clinical interventions may be helpful for patients with AN and OCD.

## Data Availability Statement

The raw data supporting the conclusions of this article will be made available by the authors, without undue reservation.

## Ethics Statement

The studies involving human participants were reviewed and approved by Medical Ethics Committee of the University Medical Center Utrecht (NL43751.041.13v0.3) and registered at the Netherlands Trial Register (NTR3865) prior to the start of data collection. The patients/participants provided their written informed consent to participate in this study.

## Author Contributions

LS, DC, and UD contributed to conception and design of the study. LS wrote the first draft of the manuscript. UD organized the database and performed the analysis. BP and AD wrote sections of the manuscript. All authors contributed to manuscript revision, read, and approved the submitted version.

## Funding

This study was supported by bij ZonMW grand 837001004. Open access publication fees paid by the Utrecht University Open Access Fund.

## Conflict of Interest

The authors declare that the research was conducted in the absence of any commercial or financial relationships that could be construed as a potential conflict of interest.

## Publisher's Note

All claims expressed in this article are solely those of the authors and do not necessarily represent those of their affiliated organizations, or those of the publisher, the editors and the reviewers. Any product that may be evaluated in this article, or claim that may be made by its manufacturer, is not guaranteed or endorsed by the publisher.
